# Oral peptide drug delivery: design of SEDDS providing a protective effect against intestinal membrane-bound enzymes

**DOI:** 10.1007/s13346-025-01852-6

**Published:** 2025-04-24

**Authors:** Annika Postina, Dennis To, Katrin Zöller, Andreas Bernkop-Schnürch

**Affiliations:** Center for Chemistry and Biomedicine, Department of Pharmaceutical Technology, Institute of Pharmacy, Leopold-Franzens-University of Innsbruck, Innrain 80/82, 6020 Innsbruck, Austria

**Keywords:** SEDDS, Peptide drugs, Membrane-bound enzyme, Oral drug delivery, Hydrophobic ion pairing

## Abstract

This study presents an emerging approach for oral peptide drug delivery by designing self-emulsifying drug delivery systems (SEDDS) capable of protecting peptide drugs from intestinal brush border membrane-bound (BBM) enzymes. Tuftsin was incorporated as a model peptide into three optimized SEDDS formulations through hydrophobic ion pairing with the anionic surfactants docusate (AOT), N-octadecyl sulfate (OS), and lauryl sulfate (LS). SEDDS- 1 consisted of octyldodecanol, polyoxyethylene (10) oleyl ether, polyoxyethylene (20) oleyl ether and citronellol. SEDDS- 2 were formulated with isopropylmyristate, polyoxyethylene (20) oleyl ether and eugenol. SEDDS- 3 included caprylic acid, PEG- 35 castor oil and citronellol. The resulting nanoemulsions were characterized for droplet size, zeta potential, polydispersity index (PDI), and stability in biorelevant media. Enzymatic degradation studies with aminopeptidase N and ex vivo rat small intestine revealed remarkable protective effects. SEDDS- 3 exhibited superior performance, preserving over 80% of tuftsin after 4 h, followed by SEDDS- 2 protecting 70% of tuftsin, while the unformulated peptide was entirely degraded within 20 min. Furthermore, SEDDS- 2 demonstrated enhanced permeation of tuftsin across intestinal mucosa by achieving a 4-fold increase, while SEDDS- 3 led to a 3-fold enhancement in permeation compared to the unformulated peptide. Accordingly, SEDDS- 3 demonstrates the greatest potential for peptide delivery due to its superior protective performance and enhanced permeation compared to the control. These findings underscore the potential of SEDDS as a versatile platform for safeguarding peptide drugs against enzymatic degradation in the intestinal environment. By modifying their composition, SEDDS can unlock new possibilities for efficient oral peptide drug delivery, overcoming critical enzymatic and mucosal barriers.

**Graphical Abstract:**

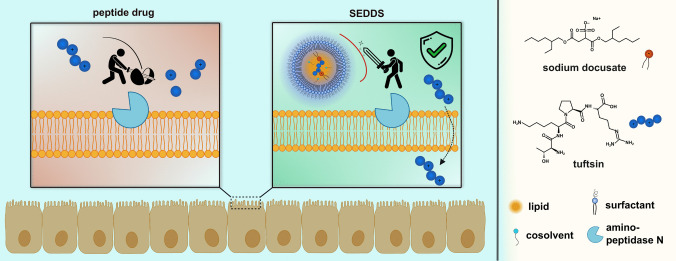

**Supplementary Information:**

The online version contains supplementary material available at 10.1007/s13346-025-01852-6.

## Introduction

Peptide drugs have been conquering the global pharmaceutical market for the treatment of diverse diseases since several decades [1]. As the prevalent parenteral administration of most peptide drugs is associated with risks and poor compliance, alternative routes of administration are of great interest. In particular oral delivery systems for these drugs are in focus of academic and industrial research being favored by patients [2]. Successful oral administration, however, faces numerous challenges including acidic conditions of the stomach as well as the mucus and epithelial barrier that have to be overcome on the route to the systemic circulation. Another barrier that peptide drugs are facing on this route is the enzymatic barrier caused by luminally secreted and membrane-bound enzymes. Strategies to overcome this barrier include for example enzyme inhibition by co-administration of enzyme inhibitors [3–5], peptide drug modifications eliminating cleavage sites and permeation enhancement [6]. Another strategy is the utilization of different types of lipid-based nanocarriers. Due to the lipophilic environment of these nanocarriers, they are inaccessible to enzymes providing protection for incorporated peptides or proteins. Self-emulsifying drug delivery systems (SEDDS) represent a promising lipid-based nanocarrier system, since they have already shown a protective effect towards gastrointestinal enzymes such as trypsin, α-chymotrypsin or pepsin [7–10]. After surpassing the stomach, the drug must be absorbed mainly via enterocytes in the small intestine in order to reach the blood stream [2]. Uptake mechanisms of lipid-based nanocarriers like endocytosis or fusion events require a direct interaction of the carrier with intestinal epithelial cells [11]. Since membrane-bound enzymes are localized at this site of action, it is of great interest to investigate whether SEDDS are capable of providing effective protection of the peptide at the membrane. Hence, it was the aim of this study to investigate the protective effect of SEDDS against brush border membrane-bound enzymes. Since aminopeptidase N is a prominent BBM enzyme being expressed on the intestinal mucosa [12] and selectively cleaving individual neutral amino acids from the N-terminus of bioactive peptides, we focused in this study in particular on this exopeptidase.

Tuftsin was chosen as model peptide since it is rapidly degraded by aminopeptidase N resulting in low bioavailability and a short half-life [13]. Stimulating phagocytosis and chemotaxis of immune cells, tuftsin holds therapeutic potential with antibiotic, anti-inflammatory and anti-carcinogenic properties [14].

SEDDS containing lipase-indigestible excipients providing high stability of the carrier itself against enzymatic degradation [11, 15] were developed. To facilitate the integration of this hydrophilic peptide into the oily droplets, its lipophilicity was enhanced through hydrophobic ion pairing. The loaded SEDDS were analyzed regarding their size and PDI in biorelevant media, along with an evaluation of their cytotoxic potential. Furthermore, the stability of the formulations against aminopeptidase N was investigated by incubation with isolated enzyme and rat intestine, measuring the residual concentration of the peptide. Subsequently, the amount of tuftsin permeating rat intestinal mucosa without being degraded because of the protective effect of SEDDS was examined.

## Materials and methods

### Materials

Tuftsin acetate was provided by Bachem AG (Bubendorf, Switzerland). Aminopeptidase N from porcine kidney, octanoic acid (caprylic acid), penicillin, streptomycin, phosphate buffered saline (PBS), trypsin and fetal bovine serum (FBS) were obtained from Merck KGaA (Darmstadt, Germany).

Isopropyl myristate, sodium lauryl sulfate (LS), 4-nitroaniline, 4-(2- hydroxyethyl)− 1-piperazineethanesulfonic acid (HEPES, ≥ 99.5%) and tris(hydroxymethyl)aminomethane (TRIS, ≥ 99.9%) were purchased from Carl Roth GmbH (Karlsruhe, Germany). 2-Octyldodecan- 1-ol was supplied by TCI (Eschborn, Germany). *L*-Leucine- 4-nitroanilide and 1-octanol were obtained from ThermoFisher GmbH (Kandel, Germany). Sodium docusate (AOT) and sodium *N*-octadecyl sulfate (OS) were provided by Alfa Aesar. FaSSIF/FaSSGF powder was obtained from Biorelevant (London, United Kingdom). Water for HPLC and *D*-glucose anhydrous (≥ 99.5%) were supplied by VWR (Linz, Austria). PEG- 35 castor oil (Kolliphor® EL), polyoxyethylene (20) oleyl ether (Brij® O20), polyoxyethylene (10) oleyl ether (Brij® O10), eugenol, citronellol, trifluoroacetic acid (TFA), acetonitrile, minimum essential medium eagle (MEM), resazurin sodium salt, Triton-X 100, potassium chloride, calcium chloride and sodium chloride were purchased from Sigma-Aldrich (Vienna, Austria).

### Tuftsin quantification via HPLC

Tuftsin quantification was carried out using RP-HPLC, following a methodology previously described [16] with some modifications. The analytical setup comprised a Chromaster 5430 diode array detector, Chromaster 5310 column oven, Chromaster 5260 auto sampler, and Chromaster 5160 pump. The stationary phase featured a Waters XSelect® HSS C18 column (4.6 × 250 mm, 5 µm) (Vienna, Austria). A binary solvent system consisting of water and acetonitrile (96:4, v/v) with 0.1% (v/v) trifluoroacetic acid (TFA) served as the mobile phase. Isocratic elution was conducted at a flow rate of 1.2 mL/min over a duration of 10 min. The column temperature was set to 40 °C and the injection volume was 20 µL. Tuftsin was detected at a wavelength of 220 nm. A calibration curve covering a concentration range from 1 to 0.008 mg/mL was established (R^2^ > 0.99).

### Preparation and characterization of SEDDS

**Fig. 1:**
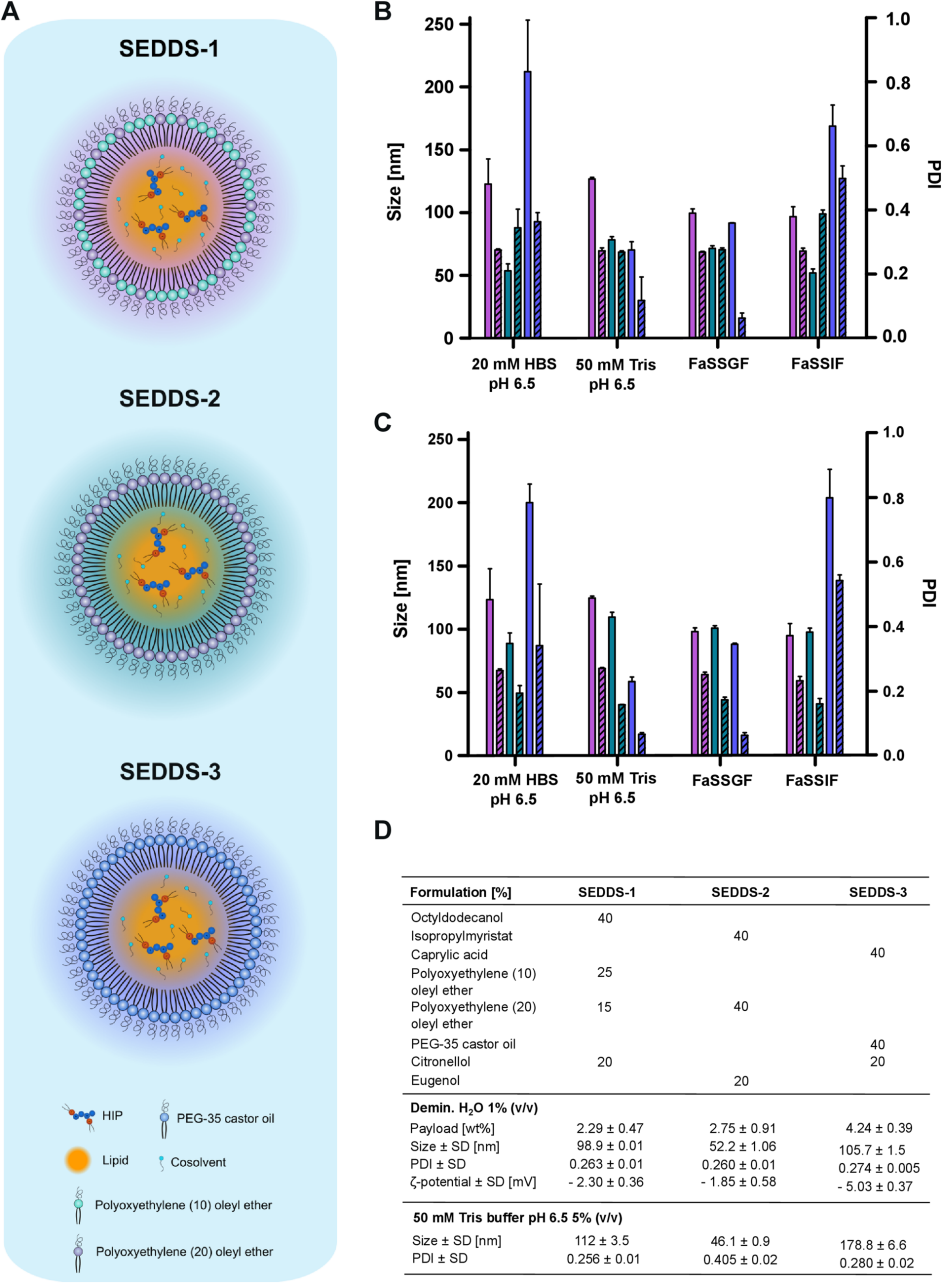
(**A**) Schematic illustration of SEDDS emulsified in aqueous medium. (**B**) Alterations of droplet size [nm]  and PDI  of 1% (v/v) SEDDS- 1  , SEDDS- 2  and SEDDS- 3  in indicated buffer systems and biorelevant media immediately after dilution and (**C**) after 4 h of incubation at 37 °C and 400 rpm. (**D**) Composition of SEDDS, their payload for tuftsin-AOT HIP, droplet size, PDI and zeta potential of 1% (v/v) SEDDS in demineralized water together with size and PDI for 5% (v/v) SEDDS in 50 mM Tris buffer pH 6.5. Indicated values are means ± standard deviation (n ≥ 3)

For the preparation of SEDDS lipids, surfactants, and cosolvents resistant to lipase were chosen to ensure sufficient stability of the formulations. Excipients as listed in Fig. 1D were homogenized using a Thermomixer at 60 °C and 2000 rpm for 2 h to produce the SEDDS preconcentrates. Post-preparation, preconcentrates were emulsified in a concentration of 1% (v/v) in demineralized water or 5% (v/v) in 50 mM Tris buffer pH 6.5 for the characterization of size and polydispersity index (PDI). Additionally, the zeta potential was determined for samples emulsified in demineralized water. The resulting nanoemulsions were preheated to 37 °C and agitated using a thermomixer before measurement.

Data were obtained via dynamic light scattering (DLS) for size measurements and electrophoretic light scattering for zeta potential using the ZetaSizer Nano ZSP (Malvern Instruments in Worcestershire, United Kingdom). All samples were analyzed in triplicates at 37 °C.

Furthermore, the stability of the nanoemulsions was determined at a concentration of 1% (v/v) in biorelevant media, including fasted state simulated gastric fluid (FaSSGF) and fasted state simulated intestinal fluid (FaSSIF) [17], as well as in 20 mM HBS pH 6.5 and 50 mM Tris buffer pH 6.5. The size and PDI of the emulsions were monitored over a 4 h time period.

HIP-loaded SEDDS for degradation studies were prepared by first dissolving the HIP in SEDDS preconcentrates through agitation on a thermomixer at 37 °C and 2000 rpm overnight. The concentrations employed for SEDDS- 1, SEDDS- 2, and SEDDS- 3 were 20 mg/mL, 26 mg/mL, and 30 mg/mL, respectively. These concentrations were derived from the maximum solubility of HIP in SEDDS preconcentrates as described in the following chapter. Subsequently, SEDDS preconcentrates containing HIP were emulsified 5% (v/v) in 50 mM Tris buffer pH 6.5 or 20 mM HBS pH 6.5.

### Hydrophobic ion pairing

Hydrophobic ion pairing was carried out to increase lipophilicity of tuftsin. To that end the peptide was dissolved in a concentration of 1 mg/mL in 0.01 M HCl. Counterions, namely AOT, OS, and LS, were dissolved in 1 mL 0.01 M HCl with molar ratios of 3:1, 4.5:1 and 6:1 corresponding to charge ratios of 1, 1.5 and 2 (counterion:tuftsin) [18, 19]. To form the complex, 500 µL of the counterion solution were added dropwise to 500 µL of the peptide solution using a Thermomixer (Eppendorf AG, Germany). The mixture was incubated at 25 °C while being agitated at 400 rpm for 1 h. Afterwards, the samples were centrifuged at 12,500 rpm for 15 min using a MiniSpin® (Eppendorf AG, Germany) [18, 20].

The resulting precipitate of tuftsin-counterion complex was washed twice with 0.01 M HCl and then lyophilized (Christ Gamma 1–16 LSC Freeze Dryer, Martin Christ Gefriertrocknungsanlagen GmbH, Germany). The obtained complex was stored at − 20 °C until further use.

### Maximum solubility and payload of ion pair in SEDDS preconcentrates

In order to determine the maximum solubility of the hydrophobic ion pair (HIP) in SEDDS preconcentrates, the latter were added to an excess of HIP and the resulting samples were agitated for 24 h at 37 °C. Following this incubation, the samples were centrifuged at 12,500 rpm for 15 min to separate undissolved HIP and 10 µL aliquots were collected from the supernatant [18]. Before injection into the HPLC system, these aliquots were diluted in a ratio of 1:9 with methanol containing 0.1% (v/v) TFA to facilitate HIP dissociation and to determine the concentration of dissolved HIP. The weight of maximum dissolved HIP corresponds to the maximum payload (wt%) of HIP in SEDDS preconcentrates [21]. Therefore, the weight of each SEDDS preconcentrate was determined by weighing 1 mL aliquots using an analytical balance (Sartorius MSE225P- 100-DI, Göttingen, Germany). Payloads were calculated by Eq. (1):

1 $$payload\;\left(wt\%\right)= \frac{w\;of\;HIP\;maximum\;dissolved\;in\;1\;mL\;SEDDS\;preconcentrate}{w\;1\;mL\;SEDDS\;preconcentrate}\;\times 100$$

### Log P_1-octanol/water_ and log D_SEDDS__/release medium_ determination

The distribution coefficient (logP) of HIPs and tuftsin between 1-octanol and water was determined. HIPs were dissolved and tuftsin was dispersed in 1-octanol. Subsequently, an equal volume of water was added to the organic phase and the samples were incubated for 24 h at 37 °C with continuous agitation at 400 rpm using a thermomixer [22]. Following the incubation period, the samples were subjected to centrifugation at 12,500 rpm for 15 min to separate the phases. The aqueous phase was directly injected into the HPLC system, while the 1-octanol phase was diluted with a mixture of 0.1% (v/v) TFA in methanol at a 1:3 ratio. LogP values were calculated by using Eq. (2):

2 $$\mathrm{log}{P}_{1-octanol/water}=log\frac{{c}_{ tuftsin\;in\;1-octanol}}{{c}_{tuftsin\;in\;water}}$$

The distribution of HIP between SEDDS and release medium was determined by quantifying the maximum solubility of HIPs in SEDDS preconcentrates and HEPES buffered saline (HBS) composed of 20 mM HEPES, 1 g/L glucose anhydrous, 136.7 mM NaCl, 5 mM KCl and 1 mM CaCl_2_. An excess amount of HIP was added to the respective medium and samples were agitated for 24 h at 37 °C [23]. After incubation, the samples were centrifuged at 12,500 rpm for 15 min and the concentration of tuftsin in the supernatant was determined. The calculation was performed using Eq. (3):

3 $$\mathrm{log}{D}_{SEDDS/HBS}=log\frac{{c}_{tuftsin\;in\;NE\;preconcentrate}}{{c}_{tuftsin\;in\;HBS}}$$

### Cell viability – resazurin assay

The potential cytotoxic properties of HIP-loaded SEDDS were investigated via resazurin assay at various SEDDS concentrations. Caco- 2 cells were seeded at a density of 5 × 10^4^ cells per well in a 96-well plate and cultured for 72 h at 37 °C, with 95% humidity and 5% CO_2_ to reach confluency. The culture medium employed was Minimum Essential Medium (MEM), containing 10% (v/v) heat-inactivated fetal bovine serum (FBS) and a penicillin/streptomycin solution (final concentration 100 units/0.1 mg/L) [24].

SEDDS were emulsified in sterile HBS at pH 7.4 at concentrations of 0.01%, 0.0125%, 0.025%, and 0.05% (v/v). A 0.1% (v/v) Triton-X solution in HBS served as a positive control, while sterile HBS applied to cells served as the negative control.

After removing MEM, cells were washed twice with prewarmed HBS. The cells were treated with SEDDS for a period of 4 h and 24 h, with an application volume of 100 µL. After the incubation period, cells were washed twice and 100 µL 0.1% (m/v) resazurin solution in HBS were added. After 2 h of incubation, aliquots of 100 µL were transferred into a black 96-well plate and fluorescence intensity was measured at an excitation wavelength of 540 nm and an emission wavelength of 590 nm with the Tecan Spark plate reader. Cell viability was calculated according following Eq. (4):

4 $$Cell\;viability\;\left(\%\right)= \frac{intensity\;of\;sample-intensity\;of\;positive\;control}{intensity\;of\;negative\;control-intensity\;of\;positive\;control} \times 100\%$$

### Degradation study by isolated aminopeptidase N

SEDDS were initially loaded with HIPs to determine the degradation of the incorporated peptide by isolated aminopeptidase N. HIPs were dissolved in preconcentrates of SEDDS at maximum solubility as previously mentioned at 37 °C and 2000 rpm overnight. Subsequently, the preconcentrates were emulsified in 50 mM Tris buffer pH 6.5, resulting in a final concentration of 5% (v/v). Tuftsin was dissolved in a concentration of 1 mg/mL in the same buffer. Aminopeptidase N suspension was diluted with 50 mM Tris buffer pH 6.5 to reach a final concentration of 0.63% (v/v) corresponding to 53.5 mU/mL of enzyme activity [25] and incubated for 15 min at 37 °C. To initiate the enzymatic reaction, samples and the enzyme solution were mixed in a 1:1 ratio and further incubated at 37 °C and 400 rpm for 4 h. At predetermined time intervals, 50 µl aliquots were withdrawn from each sample and an equal volume of methanol containing 2% (v/v) TFA was added to terminate the enzymatic reaction [26]. Following this, the concentration of tuftsin in the supernatant was quantified using HPLC.

### Degradation study with rat intestine

Rat intestine was obtained freshly from male Sprague–Dawley rats (200–300 g) supplied by Janvier Labs (Saint Berthevin, France). The middle section of the small intestine was longitudinally dissected and thoroughly washed with HBS pH 6.5 to remove intestinal contents. The intestine was stored at − 20 °C in HBS until further use.

After thawing the small intestine was cut into 1 × 1 cm pieces that were placed in tubes containing 500 µL of HBS. SEDDS with dissolved HIP were emulsified in a concentration of 10% (v/v) in HBS. 500 µL of nanoemulsions were added to each tube to obtain a final concentration of 5% (v/v) SEDDS. Thereafter, samples were incubated on a thermomixer at 37 °C with continuous agitation at 400 rpm. Over a 4 h time period, 100 µL aliquots were withdrawn from each sample at predefined time intervals. To terminate the enzymatic reaction, an equal volume of methanol containing 2% (v/v) TFA was added to each aliquot. Subsequently, the concentration of tuftsin in the supernatant was quantified using HPLC.

### Determination of aminopeptidase N enzyme activity

The enzymatic activity of rat intestine in the presence and the absence of SEDDS was determined. This was carried out to confirm enzyme activity after the freezing and thawing process and to assess any potential inhibitory effects of SEDDS on the enzyme activity of rat intestine.

*L*-Leucine- 4-nitroanilide was used as substrate, which undergoes hydrolysis by aminopeptidase N to liberate the colored product 4-nitroaniline. The substrate was dissolved in a concentration of 5 mM in 20 mM HBS pH 6.5. To determine the enzymatic activity of rat intestinal mucosa, 1 × 1 cm pieces were placed in 500 µL of 20 mM HBS pH 6.5 mixed with 500 µL of the substrate solution and then incubated at 37 °C with continuous agitation at 400 rpm for 4 h. Afterwards, 500 µL aliquots were withdrawn from each sample and diluted with methanol containing 2% (v/v) TFA before centrifugation at 12,500 rpm for 5 min. The absorbance of the supernatant (100 µL) was measured photometrically at a wavelength of 405 nm by Tecan Spark (Tecan Sales Austria GmbH, Austria) [27]. The concentration of the colored product was determined using a calibration curve with 4-nitroaniline in HBS in the concentration range of 69–2.15 µg/mL.

To investigate potential inhibitory effects of SEDDS on aminopeptidase N activity on rat intestinal mucosa, 1 × 1 cm pieces of rat intestinal mucosa were incubated with each 500 µL of HBS, substrate solution and 5% (v/v) SEDDS. After 4 h of incubation at 37 °C and 400 rpm agitation, aliquots were withdrawn from each sample and appropriately diluted with methanol containing 2% (v/v) TFA to dissolve all contents. After centrifugation at 12,500 rpm for 5 min, the amount of 4-nitroaniline in 100 µL of the supernatant was quantified at 405 nm. Enzyme activity was calculated in mU, assuming that 1 U converts 1 µmol substrate/min.

Furthermore, a possible inhibitory effect of SEDDS and excipients on aminopeptidase N activity was investigated using the isolated enzyme, following a method previously described with slight modifications [28]. Surfactant solutions were prepared in 20 mM HBS pH 6.5 in the same concentration as they are present in SEDDS. Either 5% (v/v) SEDDS or surfactant solution were mixed in 500 µL together with further 500 µL of a 41.6 mU/mL enzyme solution in 20 mM HBS pH 6.5. *L*-Leucine- 4-nitroanilide was added prior to SEDDS and surfactant solution in a final concentration of 5 mM.

A 5 mM substrate solution served as the positive control, representing 100% enzyme activity. The samples were incubated on a thermomixer for 1 h at 37 °C and 400 rpm agitation. To stop the enzymatic reaction, 1 mL of methanol containing 2% (v/v) TFA were added to each sample. After centrifugation at 12,500 rpm, the concentration of 4-nitroaniline in the supernatant was quantified. The inhibitory activity was calculated using the following Eq. (5):

5 $$Inhibitory\;activity\;[\%]=100-\left( \frac{{Abs}_{sample}}{{Abs}_{positive\;control}}\right)\times100$$

### Ex-vivo permeation study on rat intestinal mucosa

The amount of permeated peptide was determined via an ex vivo permeation study on freshly excised mucosa from Sprague–Dawley rats, following a previously established protocol with some modifications [29]. The rat intestine was carefully cleaned to remove its intestinal contents and then cut into 1.5 cm sections. These sections were fixed in Ussing chambers with a surface area of 0.64 cm^2^ and 1 mL of 20 mM HBS pH 6.5 was added to each acceptor chamber, while the donor chambers were filled with 1 mL of 5% (v/v) SEDDS in 20 mM HBS pH 6.5, facing the luminal side of the intestine.

The Ussing chambers were incubated in a water bath at 37 °C, and every 1 h, 100 µL aliquots were withdrawn from the acceptor chambers over a 4 h period. Withdrawn samples were replaced with pre-warmed HBS to maintain sink conditions. The concentration of permeated tuftsin in the collected aliquots was determined via HPLC and the apparent permeability coefficient [30]was subsequently calculated using the following Eq. (6):

6 $${P}_{app}[cm {s}^{-1}]= \frac{Q [\mu g]}{A \left[{cm}^{2}\right] c \left[\mu g {mL}^{-1}\right] t [s]}$$

where P_app_ is the apparent permeability coefficient, Q is the total amount of test substance permeated through the mucosa, A is the diffusion area of the Ussing chamber system, c is the initial concentration of tuftsin in the donor compartment and t is the total time of experiment.

### Statistical design and data analysis

All experiments were performed at least in triplicate and results were presented as means ± standard deviation. Statistical data analysis was performed using the student t-test and the one-way ANOVA (GraphPad Prism 5) followed by Bonferroni correction with p ≤ 0.05 as the minimal level of significance.

## Results and discussion

### Characterization and stability of SEDDS

All formulations exhibited droplet sizes ranging from 46 to 180 nm in a concentration of 1% (v/v) in demineralized water and in a concentration of 5% (v/v) in 50 mM Tris buffer pH 6.5. These droplet sizes as illustrated in Fig. 1B-D are particularly suitable for oral administration, allowing the nanocarrier to easily cross the mucus gel layer and to reach the absorption site of the intestinal mucosa [11]. The PDI of < 0.3 ensures a narrow droplet size distribution and confirms the stability of all formulations [31]. Only in case of SEDDS- 2 PDI increased to 0.4 at elevated concentrations indicating a broader size distribution. Stability studies in 20 mM HBS pH 6.5 and 50 mM Tris buffer pH 6.5 as well as in the biorelevant media FaSSGF and FaSSIF revealed droplet sizes ≤ 200 nm and PDI ≤ 0.3 for all formulations after 4 h of incubation, except for SEDDS- 3 in FaSSIF medium as illustrated in Fig. 1C. The slightly increased droplet size and PDI of > 0.4 for SEDDS- 3 can be attributed to the deprotonation of caprylic acid at intestinal pH that may affect droplet size and distribution [18]. Nonetheless, the increase in negative charges is leading to electrostatic repulsion which has a positive effect on the stability of emulsions. During the 4 h incubation, droplet size of SEDDS- 1 and SEDDS- 3 remained constant in all media, while they increased slightly in case of SEDDS- 2. The PDI of SEDDS- 1 remained constant during the same period, while it decreased for formulation SEDDS- 2.

**Fig. 2:**
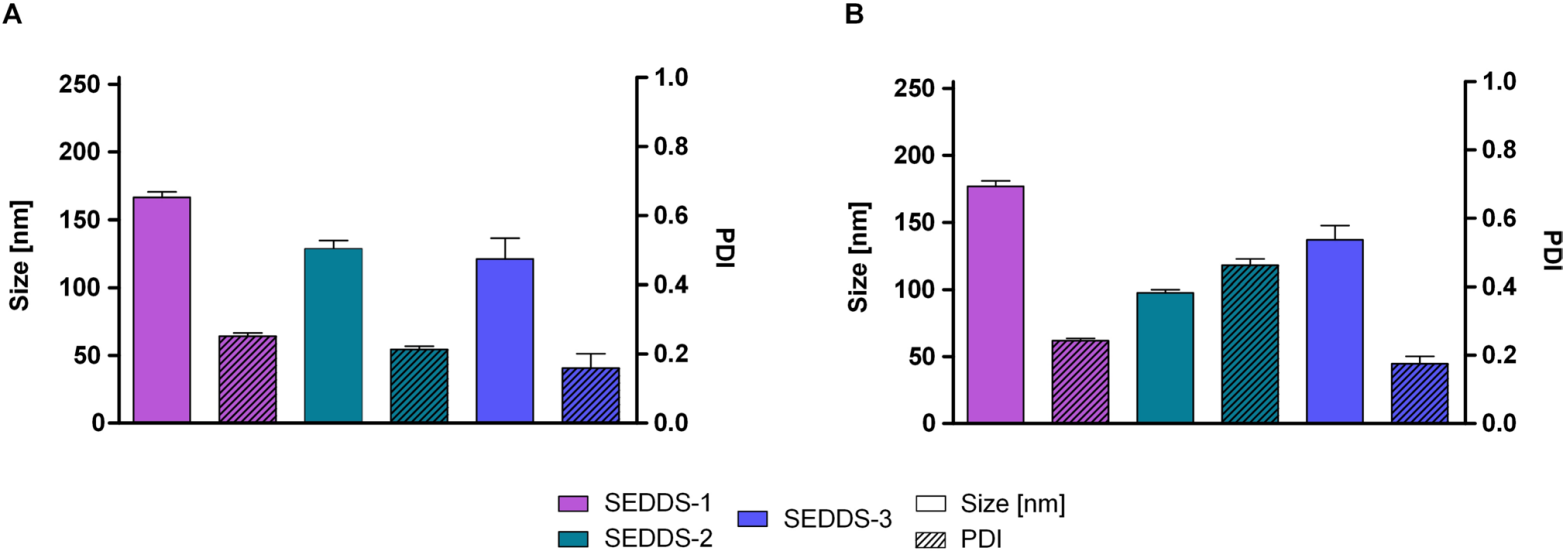
(**A**) Alterations of size (nm) and PDI of 5% (v/v) SEDDS in 50 mM Tris buffer pH 6.5 containing aminopeptidase N emulsified and (**B**) after 4 h of incubation at 37 °C and 400 rpm. Data are presented as means ± standard deviation (n ≥ 3)

Furthermore, during incubation with aminopeptidase N, all emulsions remained stable with droplet sizes ranging from 120 to 170 nm and PDIs ≤ 0.25 after the 4 h time period (Fig. 2A-B). Therefore, the addition of enzyme does not affect the stability of emulsions.

### Hydrophobic ion pairing

**Fig. 3:**
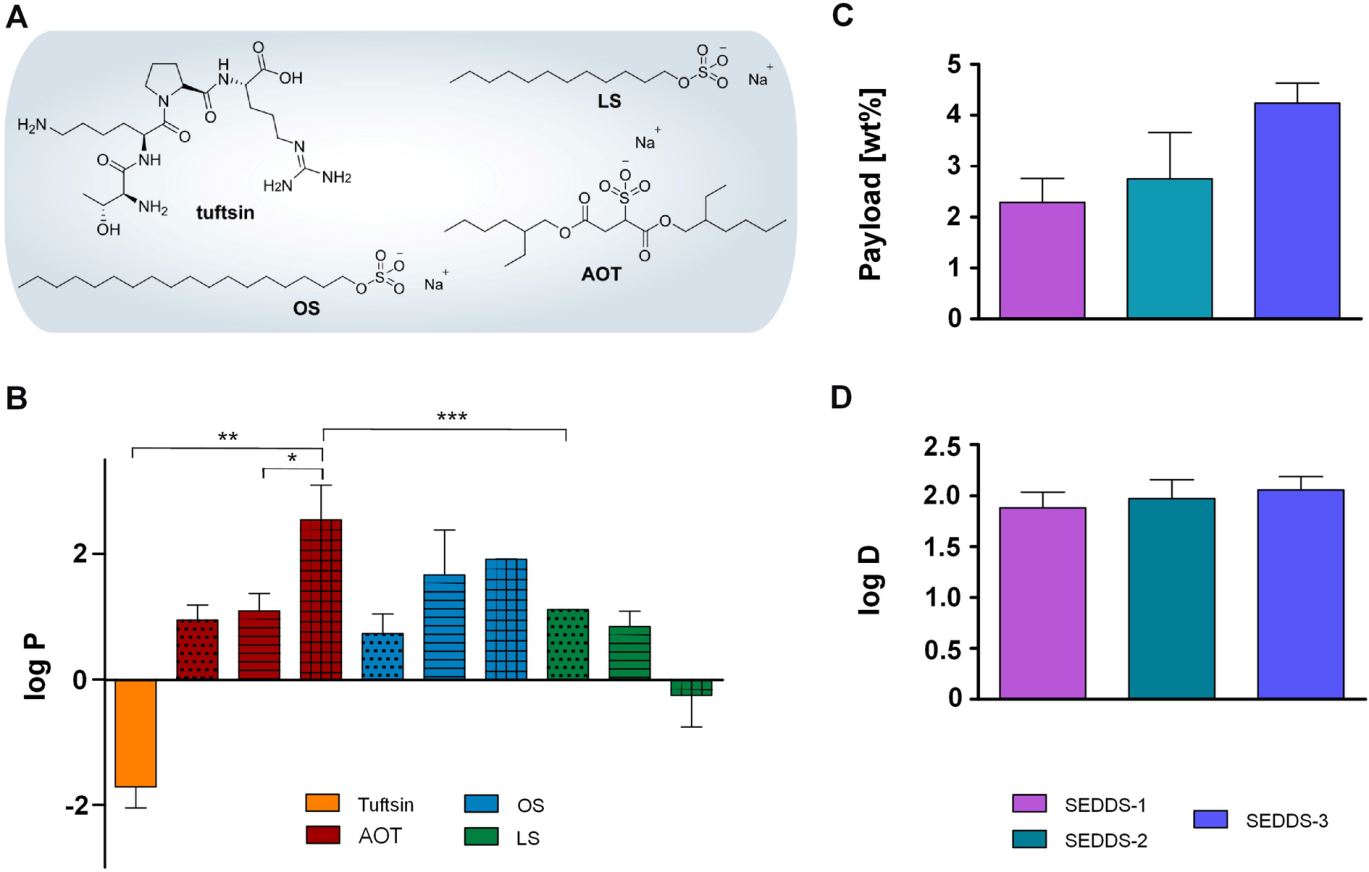
(**A**) Peptide and surfactants used for hydrophobic ion pairing. (**B**) Log P _octanol/water_ of tuftsin and HIP between tuftsin and indicated anionic surfactants in charge ratios 1  , 1.5  and 2  . (**C**) Payload of tuftsin-AOT HIP in charge ratio 2 in preconcentrates of SEDDS. (**D**) Respective logD_SEDDS/HBS_ of HIP. Data are presented as means ± standard deviation (n ≥ 3). Significant differences are indicated as * p < 0.5; ** p < 0.01; *** p < 0.001.

To increase the lipophilicity of the peptide for incorporation into SEDDS, hydrophobic ion pairing was performed. Since tuftsin bears two positively charged amino groups, various anionic surfactants were tested as counterions to form lipophilic complexes [32]. The logP values between 1-octanol and water of ion pairs having been obtained with various surfactants applied in different charge ratios were assessed as illustrated in Fig. 3B. In the case of HIPs formed with AOT and OS, logP was increasing with higher charge ratios. For LS HIPs the logP decreased with increasing charge ratio. This may be attributed to its lower carbon atom count compared to the other surfactants that can lower the hydrophobic interaction within a lipid phase. Beyond a charge ratio of 1, elevated surfactant concentrations may result in the formation of micelles, depending on the critical micelle concentration (CMC) of the surfactant. These micelles have the potential to disintegrate the HIP complex, thereby compromising its lipophilicity [32–34]. The greatest improvement in lipophilicity was achieved through the formation of the tuftsin AOT complex at a charge ratio of 2. The logP value for tuftsin substantially increased from − 1.71 to + 2.54. Hence this complex was used for further experiments.

As depicted in Fig. 3C, the tuftsin AOT complex exhibited notably high solubility across all three SEDDS formulations. The HIP was soluble in SEDDS- 1 at 20 mg/ml, SEDDS- 2 at 26 mg/ml and SEDDS- 3 at 30 mg/ml. This corresponds to payloads of 2.29%, 2.75%, and 4.24% (wt%) in SEDDS- 1, − 2 and − 3 preconcentrates, respectively. These payloads are comparably high in comparison to other studies on hydrophobic ion pairing with peptides [22, 35, 36].

High payloads are achieved by high lipophilicity of the complex and consequently high solubility in the formulation. All formulations exhibited a log D around 2, indicating the robust integration of the HIP within the oily droplets of the emulsion (Fig. 3D) [37].

### Degradation by aminopeptidase N

**Fig. 4:**
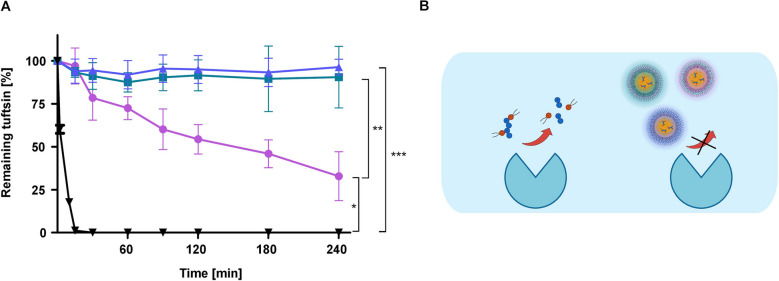
(**A**) Degradation profile of tuftsin solution  and tuftsin-AOT loaded SEDDS- 1  , SEDDS- 2  and SEDDS- 3  5% (v/v) emulsified in 50 mM Tris buffer pH 6.5 and incubated with 53.5 mU/mL aminopeptidase N. (**B**) Schematic illustration of enzymatic degradation by aminopeptidase N. Data are presented as means ± standard deviation (n ≥ 3). Significant differences are indicated as * p < 0.5; ** p < 0.01; *** p < 0.001

Previous studies have focused on the stability of SEDDS towards digestive enzymes secreted by the pancreas and their capability to protect peptides from enzymatic degradation [7–10]. After oral administration, SEDDS further face enzymes bound to the brush border membrane in the small intestine once they pass the intestinal lumen. This direct contact with membrane-bound enzymes in turn poses an additional risk of peptide digestion [38]. Hence, investigating the stability of SEDDS in the presence of BBM enzymes and protection against this kind of enzymatic degradation, is of great interest. The degradation profile of tuftsin and tuftsin loaded SEDDS during incubation with isolated aminopeptidase N is depicted in Fig. 4A.

**Fig. 5:**
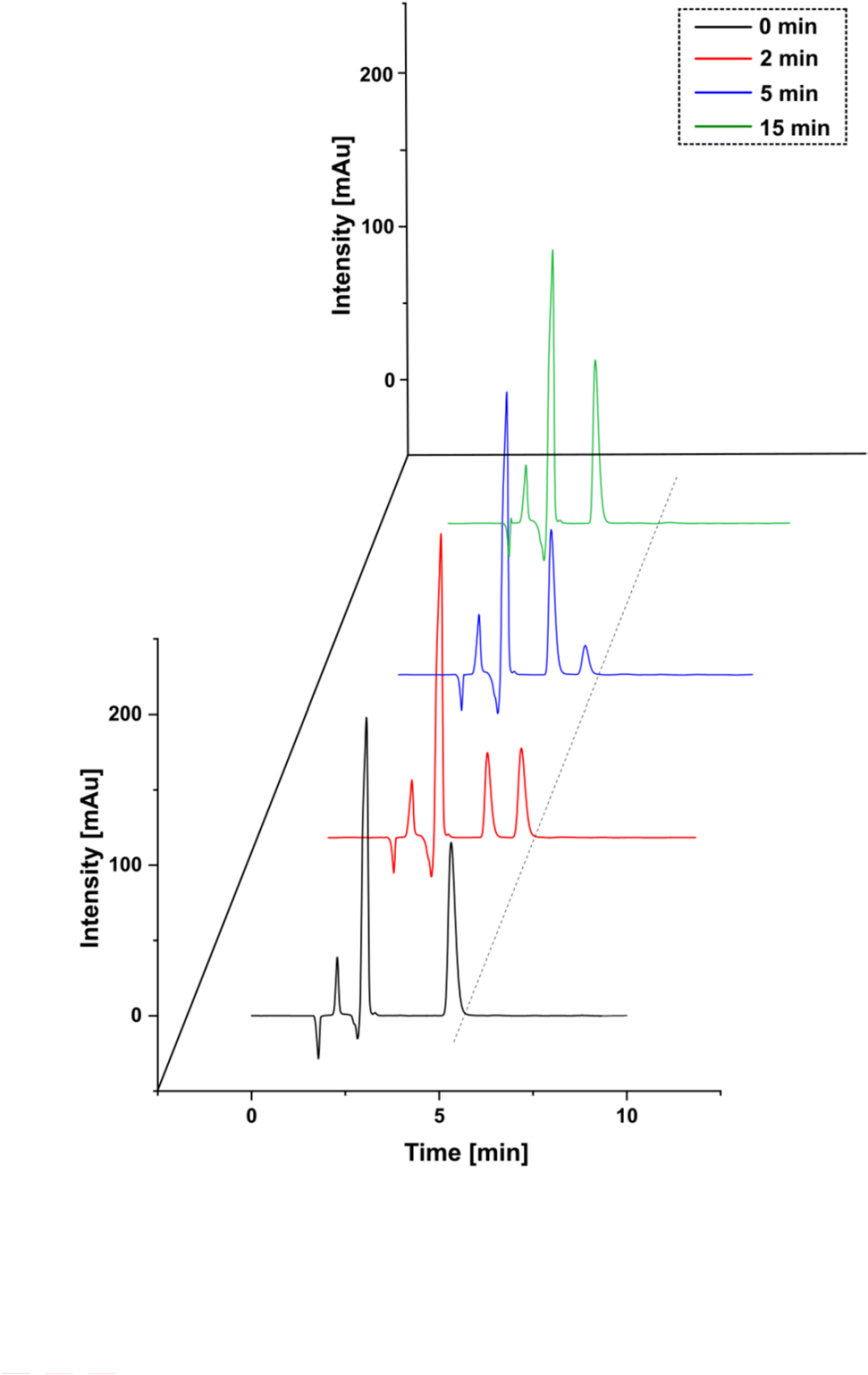
HPLC chromatogram of tuftsin dissolved 1 mg/mL in 50 mM Tris buffer pH 6.5  and degradation peak profiles of tuftsin with isolated aminopeptidase N (53.5 mU/ml) in 50 mM Tris buffer pH 6.5 at 37 °C throughout 15 min. Degradation is shown after 2 min  , 5 min  and 15 min

Tuftsin was completely degraded within 15 min in the control solution which was also confirmed by the chromatograms shown in Fig. 5. The original peak of tuftsin at a retention time of 5.3 min diminished, while a new peak with a retention time of 4.3 min emerged, which can be assigned to the cleavage product. In contrast to the control, over 90% of tuftsin remained intact in both SEDDS- 2 and SEDDS- 3 after 4 h of incubation with aminopeptidase N, with no significant difference observed between the two formulations. Both formulations effectively protected the encapsulated peptide from enzymatic degradation. In the case of SEDDS- 1, 50% of tuftsin was still present after 2 h of incubation decreasing to 30% after 4 h. Although this emulsion exhibited the least protective effect among the formulations, we still observed a 3-fold higher stability of tuftsin compared to the control. These differences among SEDDS in terms of protection against the enzyme can be explained by the different choice of excipients. SEDDS limit the access of hydrophilic enzymes and thus protect incorporated peptides due to their lipophilicity. This process is also influenced by the different lipophilic properties of the excipients used [9, 39].

**Fig. 6:**
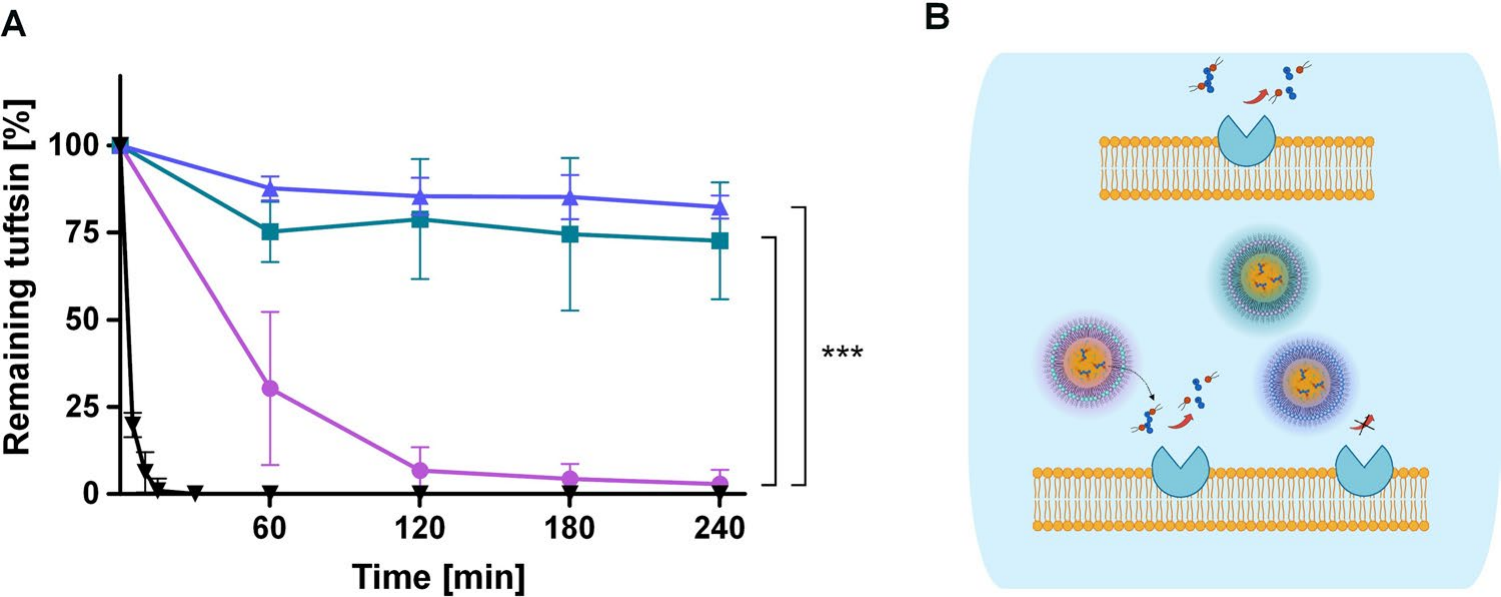
(**A**) Degradation profile of tuftsin solution  and tuftsin-AOT loaded SEDDS- 1  , SEDDS- 2  and SEDDS- 3  5% (v/v) emulsified in 20 mM HBS pH 6.5 on rat intestinal mucosa. (**B**) Schematic illustration of enzymatic degradation by membrane-bound aminopeptidase N. Data are presented as means ± standard deviation (n ≥ 3). Significant differences are indicated as * p < 0.5; ** p < 0.01; *** p < 0.001

Under conditions approximating the rat intestine, SEDDS- 2 and SEDDS- 3 provided comparable high-level protection for the peptide. After incubation on rat intestinal mucosa > 75% of undegraded tuftsin remained in both formulations (Fig. 6A). Taking into account the log D of 2 for HIP dissolved in SEDDS- 2 and SEDDS- 3 along with the Nernstsche distribution law, 95.2% of the peptide is distributed in the oil droplets of the SEDDS, which strongly supports the protection of the remaining peptide as well as the ability of HIPs to reduce premature drug release [37, 40]. In addition to the protective effect of the formulation itself, the ion pair of the peptide seems to hinder the access of peptidases [41–43]. It is therefore conceivable that the structure of the counterion is able to shield the cleavage sites of the peptide and thus provides additional protection. In case of SEDDS- 1 degradation was much faster. Nearly 80% of tuftsin in SEDDS- 1 was metabolized after 60 min, and just 2% of tuftsin remained after 4 h.

This indicates that the protective effect of the nanoemulsion is influenced by the particular composition of the formulation besides the log D and incorporation of HIP. It is also possible that the dissolved HIP in the case of SEDDS- 1 might be more surface-oriented on the oily droplets, making it more susceptible to enzymatic attack. However, it should be noted that SEDDS are also known to improve the cellular uptake of incorporated drugs [24, 44, 45]. This property could have an impact on the protective effect, as the contact time with enzymes on the membrane is shortened by the improved and thus accelerated uptake.

### Effects on aminopeptidase N enzymatic activity

**Fig. 7:**
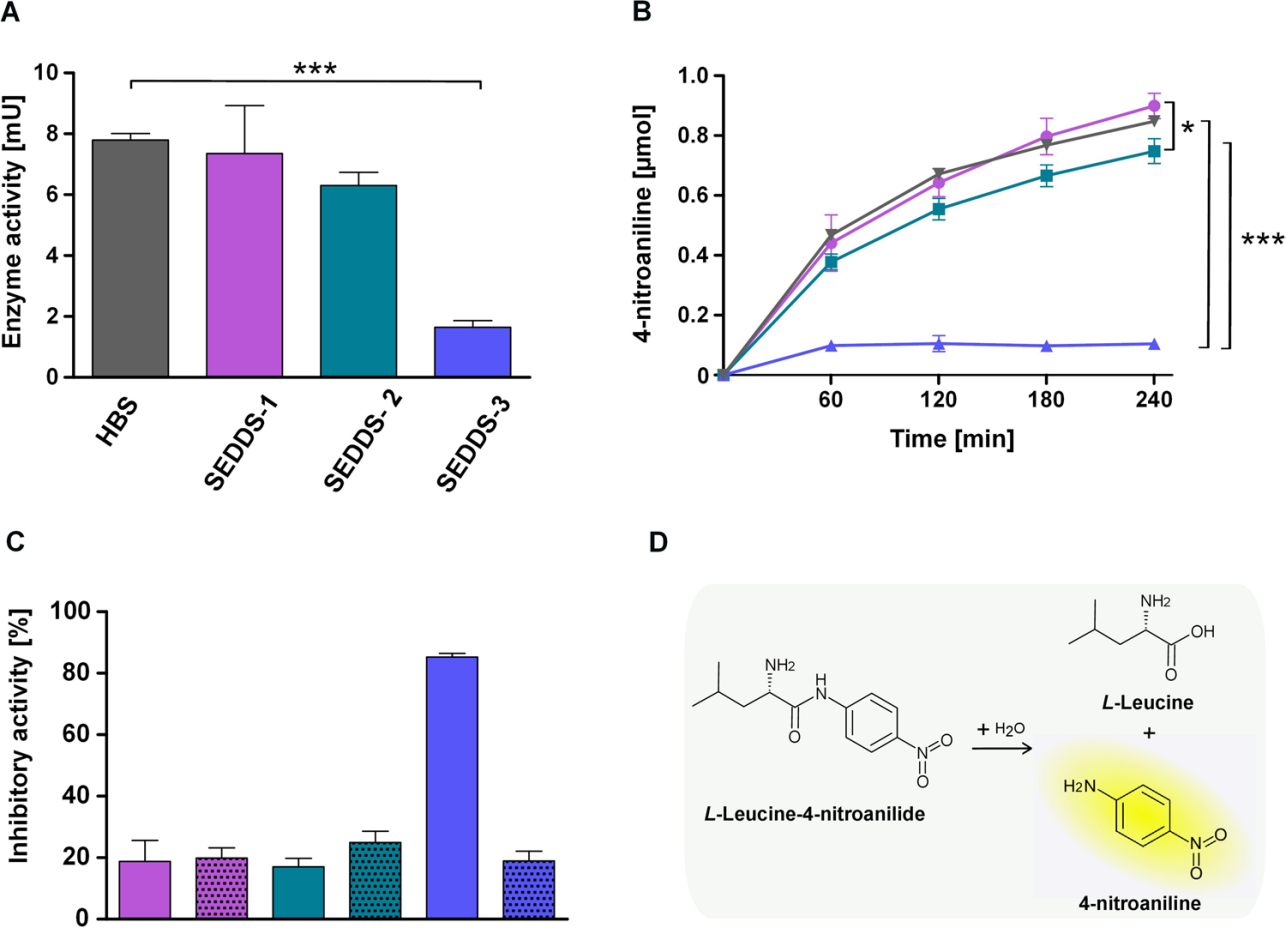
(**A**) Aminopeptidase N activity on rat intestinal mucosa after 60 min of incubation with 20 mM HBS pH 6.5 as control and with SEDDS emulsified 5% (v/v) in 20 mM HBS pH 6.5. (**B**) Formation of 4-nitroaniline on rat intestinal mucosa over 4 h of incubation with control  , SEDDS- 1  ), SEDDS- 2  and SEDDS- 3  . (**C**) Inhibitory activity of SEDDS and their corresponding surfactants  towards aminopeptidase N (**D**) Enzymatic cleavage of *L*-Leucine- 4-nitroanilide to *L*-Leucine and 4-nitroaniline. Data are presented as means ± standard deviation (n ≥ 3). Significant differences are indicated as * p < 0.5; ** p < 0.01; *** p < 0.001

To ensure the integrity and activity of aminopeptidase N on rat intestinal mucosa, both after freezing and thawing and during incubation with nanoemulsions, enzymatic activity was monitored using* L*-Leucine- 4-nitroanilide as substrate. As shown in Fig. 7A, activity of aminopeptidase N on the mucosa was nearly 8 mU/cm^2^ for the control solution with 20 mM HBS pH 6.5, aligning with findings from earlier studies on aminopeptidase N activity [46]. Incubation with SEDDS- 1 and SEDDS- 2 marginally affected enzymatic activity, while for SEDDS- 3, enzymatic activity decreased to 2 mU/cm^2^. This was shown by the formation of 4-nitroaniline illustrated in Fig. 7B. The control solution, SEDDS- 1 and SEDDS- 2 exhibited no significant differences in the formation of an average of 0.8 µmol 4-nitroaniline, whereas 0.1 µmol was formed during the incubation with SEDDS- 3 for 60 min. Hence SEDDS- 3 might exhibit an inhibitory effect on enzymatic activity of aminopeptidase N. Consequently, a more detailed investigation was conducted to determine the inhibitory properties of SEDDS. The corresponding surfactants were examined regarding their possible inhibitory effect [47, 48]. SEDDS- 1 and SEDDS- 2 showed a 20% inhibitory effect on the enzymatic activity of isolated aminopeptidase N with no significant difference to the corresponding surfactant used alone. In contrast, SEDDS- 3 demonstrated an 85% inhibition of the enzyme, while the corresponding surfactant exhibited only a 20% inhibitory activity (Fig. 7C). This suggests that the inhibitory effect cannot be solely attributed to the surfactant but is likely influenced by other components of the formulation, such as the lipid or co-solvent. The lipid component of SEDDS- 3 caprylic acid demonstrated an inhibitory activity of 80%, while citronellol exhibited only 18% of inhibitory activity (Fig. S2). These findings underscore the potential in the choice of excipients for SEDDS. Therefore, SEDDS- 3 not only provides a high level of protection for the peptide but also demonstrates a substantial inhibitory effect on the enzyme mainly driven by caprylic acid.

### Cell viability

**Fig. 8:**
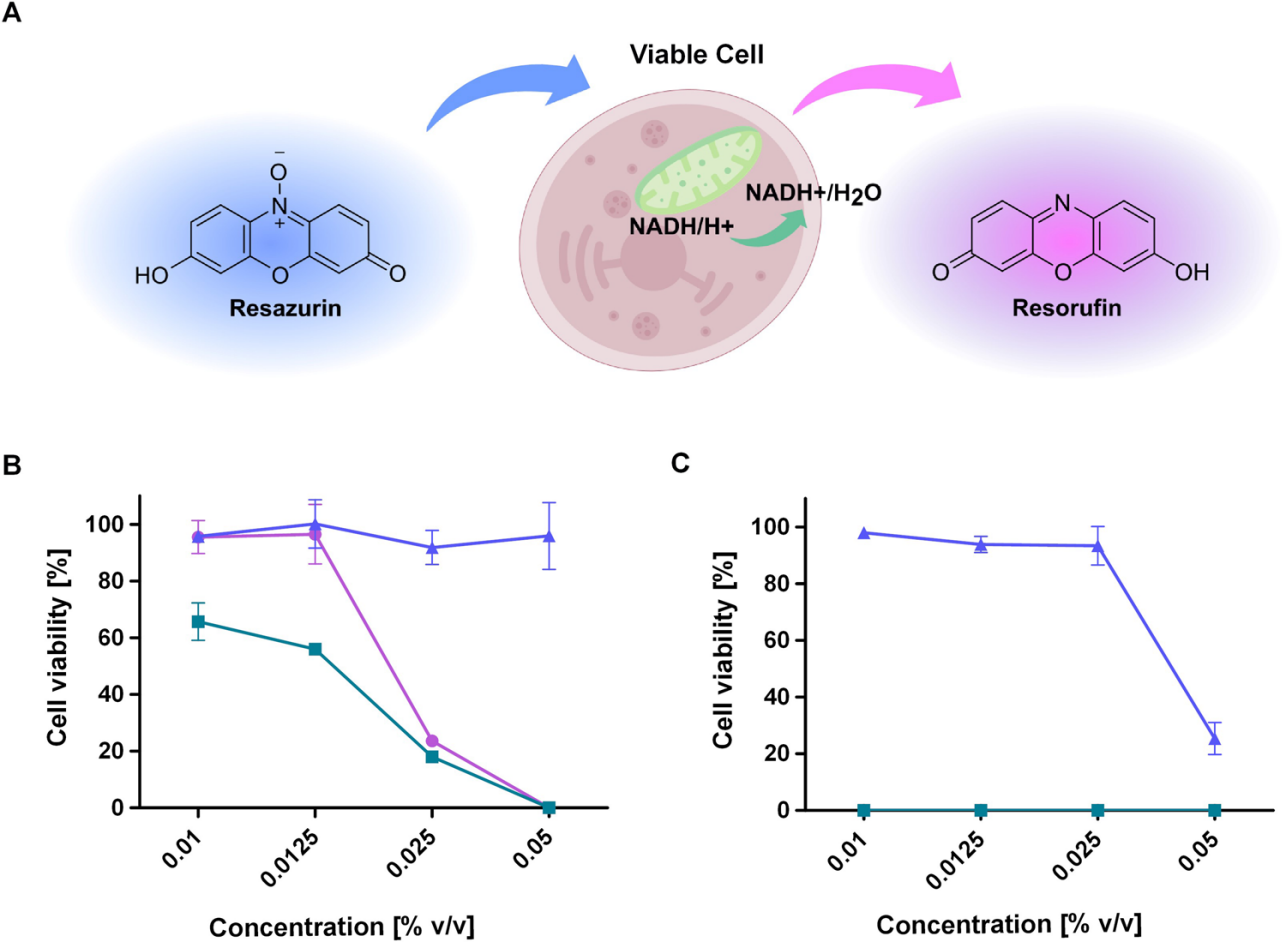
(**A**) Schematic illustration of viable cells reducing resazurin to resofurin within the resazurin assay. Viability (%) of Caco- 2 cells after 4 h (**B**) and 24 h (**C**) treatment with tuftsin-AOT loaded SEDDS- 1  , SEDDS- 2  and SEDDS- 3  . Data are presented as means ± standard deviation (n ≥ 3)

The safety of nanoemulsions was evaluated regarding cytotoxicity on Caco- 2 cell line. Since Caco- 2 cells are able to differentiate into a monolayer with properties comparable to enterocytes, this cell line was chosen to simulate conditions of the small intestine [49]. The results of the 4 h and 24 h resazurin assays are presented in Fig. 8. Cell viability was determined by calculating the percentage of living cells that reduce resazurin to its fluorescent resofurin [50]. With a minimum required cell viability of 80%, SEDDS- 3 can be categorized as non-toxic [51].

Over the entire concentration range of 0.01%—0.05% (v/v), more than 90% of cells survived after 4 h treatment with SEDDS- 3. SEDDS- 1 exhibited cell viabilities over 90% for the lower concentrations of 0.01% and 0.0125% (v/v) but showed ≤ 23% of cell viability for higher concentrations after 4. For SEDDS- 2, a concentration dependent decrease in cell viability was observed within 4 h with viability at 65% for the concentration of 0.01% (v/v) decreasing to ≤ 20% for higher concentrations of 0.025% and 0.05% (v/v). Throughout the 24 h assay, only SEDDS- 3 remained non-toxic, maintaining a cell viability of ≥ 90% for concentrations ranging from 0.01% to 0.025% (v/v). SEDDS- 2 and SEDDS- 1 showed no cell viability within the same timeframe. SEDDS- 3 thus exhibits the lowest toxic potential even over extended periods of time and can be regarded as the safest formulation. The polyoxyethylene oleyl ether surfactants utilized in SEDDS- 1 and SEDDS- 2 seem to exhibit a greater toxic potential compared to PEG- 35 castor oil, which was used for SEDDS- 3. The increased toxicity of polyoxyethylene oleyl ether surfactants relative to other PEGylated surfactants has already been observed in other studies [52–54]. Higher concentrations of nanoemulsions were used for experiments performed on rat intestine. Intestine exhibits greater resilience to external influences compared to enterocytes alone due to its nature, mucus and enzymatic as well as microbial environment. Therefore, comparatively high concentrations of nanoemulsions can be used in ex vivo permeation studies [55–57].

### Permeation on rat intestine

**Fig. 9:**
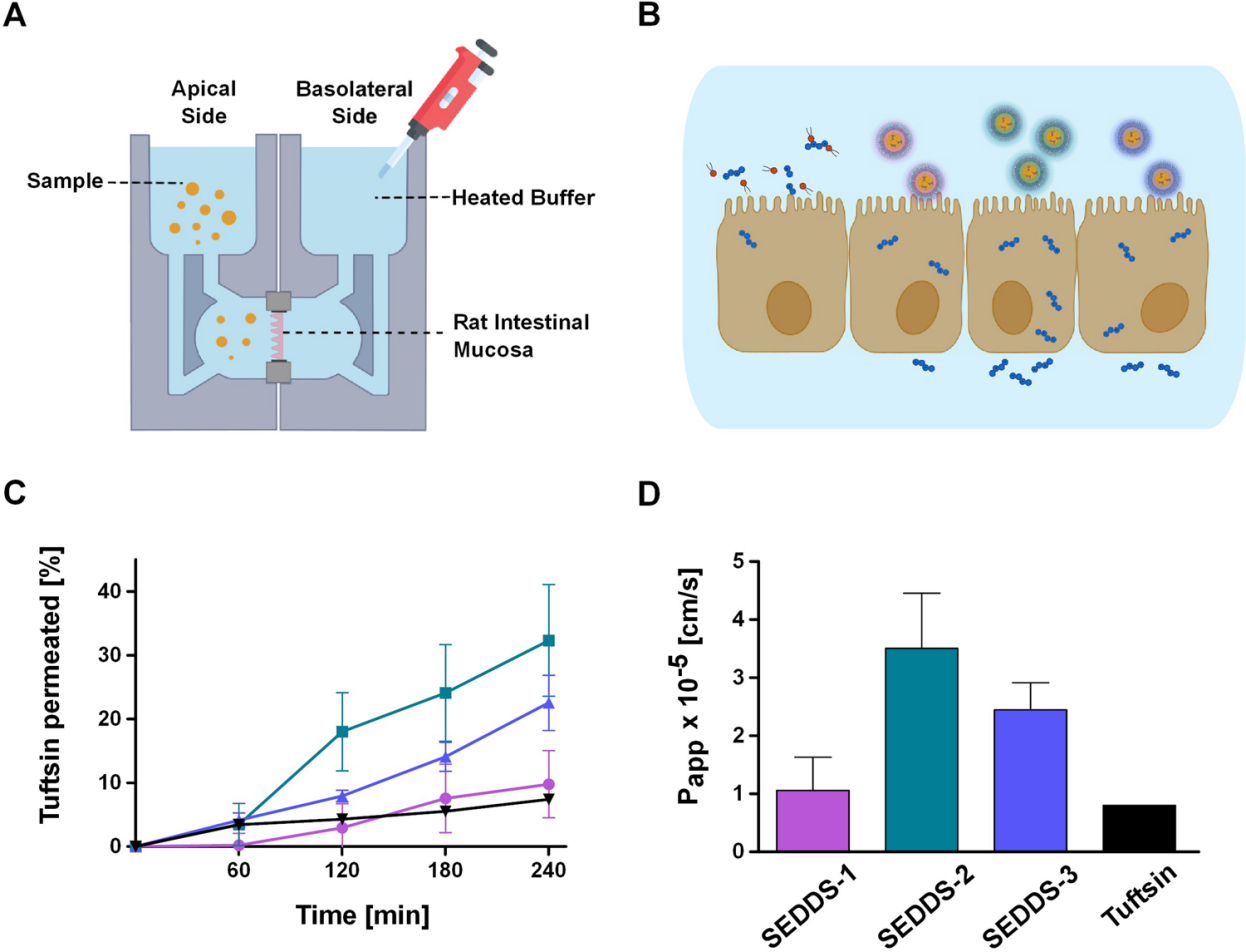
(**A**) Schematic illustration of experimental set up utilized for ex vivo permeation studies. (**B**) Schematic illustration of assumed permeation behavior of tuftsin and SEDDS. (**C**) Permeation behavior of tuftsin  and tuftsin-AOT loaded SEDDS- 1  , SEDDS- 2  and SEDDS- 3  5% (v/v) emulsified in 20 mM HBS pH 6.5 on rat intestinal mucosa. (**D**) Comparison of the apparent permeability coefficient (P_app_) of tuftsin and tuftsin-AOT loaded SEDDS on rat intestinal mucosa. Indicated values are means ± standard deviation (n ≥ 3)

The impact of SEDDS on the permeation of tuftsin was examined in the ex vivo study on freshly excised rat intestinal mucosa. Figure 9 displays the results, presenting the percentage of permeated tuftsin in the formulations alongside their corresponding permeability coefficients for tuftsin. After 4 h of cumulative permeation only 7% of initial tuftsin in the control solution permeated. Tuftsin encapsulated in SEDDS- 1 exhibited a permeation of 10%. SEDDS- 2 formulation demonstrated the highest permeation with 32%, followed by SEDDS- 3 with 22%. Since SEDDS protect tuftsin from enzymatic degradation, undegraded tuftsin is able to permeate through rat intestinal mucosa. This trend is also reflected by the calculated permeation coefficient.

When tuftsin was incorporated into SEDDS- 1, the P_app_ increased from 0.8 × 10^–5^ cm/s to 1.1 × 10^–5^ cm/s and further elevated to 2.5 × 10^–5^ cm/s for SEDDS- 3. The formulation with the highest permeation coefficient was SEDDS- 2 reaching 3.5 × 10^–5^ cm/s (Fig. 9D). This formulation provided a 4-fold higher permeation of tuftsin. The superior performance of SEDDS- 2 may be attributed to the excipients isopropyl myristate and polyoxyethylene (20) oleyl ether, both of which have been shown to enhance permeation in previous studies [58–60]. The primary function of permeation enhancing excipients to increase permeability is based on opening of epithelial tight junctions or perturbing membranes to increase flux [61]. While caprylic acid, present in SEDDS- 3, is also known to act as a permeation enhancer, it is conceivable that isopropyl myristate may provide greater permeation enhancement due to its long-chain structure. The incorporation into the membrane appears to be more effective for molecules with longer chain lengths. Consistent with this, Ruan et al. found that long-chain permeation enhancers have greater permeation enhancement efficacy than their short-chain counterparts [62]. Incorporating the peptide into SEDDS facilitates its passage across the mucosa in intact form. This also enhances the absorption of the active ingredient into the bloodstream, as the peptide remains protected from the enzymes bound to the cell membrane.

## Conclusion

The protective effect of SEDDS against the enzymatic degradation of incorporated peptide drugs by pancreatic enzymes has already been demonstrated in several studies [9, 63–65]. Our study focused on the potential of SEDDS to protect peptide drugs towards enzymatic degradation by brush border membrane-bound enzymes like aminopeptidase N. Therefore, tuftsin was incorporated as a model drug into different kind of SEDDS. This study shows that SEDDS provide a high level of protection against membrane-bound enzymes, both against isolated enzyme and on rat intestinal mucosa. The degree of protection against enzymatic degradation depends largely on the composition of the excipients in the formulation. Due to their lipophilicity, SEDDS restrict the access of hydrophilic enzymes to the integrated peptide. This also depends on the choice of excipients, which means that not every formulation appears to offer sufficient protection in this respect. The same applies to enzyme inhibition, which can be triggered by various excipients. In our study, the degree of protection increased in the following order: SEDDS- 1 < SEDDS- 2 < SEDDS- 3, with SEDDS- 3 showing 80% enzyme inhibition compared to 20% of the other SEDDS. Thus, the excipients used in this formulation, namely caprylic acid, PEG- 35 castor oil and citronellol, are of great advantage for the protection of a loaded peptide. As a result of high retention of the intact peptide due to protection towards brush border membrane-bound enzymes, SEDDS might also increase the amount of peptide that permeates the rat intestinal mucosa. This was demonstrated in the ex vivo permeation study on rat intestinal mucosa, in which a 3– to 4-fold increase in permeation was achieved by loading the peptide into SEDDS. In summary, this study outlines that SEDDS not only provide a protective effect towards membrane-bound enzymes in the intestine, but also facilitate enhanced permeation through intestinal mucosa. By overcoming the enzymatic and mucosal barrier, a greater amount of the peptide will reach its target site. Consequently, the bioavailability of such active ingredient can be substantially enhanced by utilizing the protective properties of SEDDS.

In a broader prospect, the encouraging performance of SEDDS designed in this study may be further investigated in future in vivo studies to evaluate the potential for increased bioavailability of tuftsin. This might pave the way for the oral administration of tuftsin as a therapeutically effective peptide.

## Supplementary Information

Below is the link to the electronic supplementary material.

Supplementary file1 (DOCX 189 KB)

## Data Availability

The datasets generated during and/or analyzed during the current study are available from the corresponding author on reasonable request*.*
