# Supplementary material for: Oral peptide drug delivery: design of SEDDS providing a protective effect against intestinal membrane-bound enzymes
Source: Drug Deliv Transl Res. 2025 Apr 24;16(7):2115–29. doi: 10.1007/s13346-025-01852-6 (PMC13294191; doi:10.1007/s13346-025-01852-6)
Supplement: Supplementary file 1 — Supplementary file1 (DOCX 189 KB) [file 13346_2025_1852_MOESM1_ESM.docx]

**Supplementary data**

Oral peptide drug delivery: Design of SEDDS providing a protective effect against intestinal membrane-bound enzymes

Annika Postina^1^, Dennis To^1^, Katrin Zöller^1^, Andreas Bernkop-Schnürch^1^*

^1^*Center for Chemistry and Biomedicine,* *Department of Pharmaceutical Technology, Institute of Pharmacy, Leopold-Franzens-University of Innsbruck, Innrain 80/82, 6020 Innsbruck, Austria*

*Corresponding author:

Center for Chemistry and Biomedicine, Department of Pharmaceutical Technology, Institute of Pharmacy, Leopold-Franzens-University of Innsbruck, Innrain 80/82, 6020 Innsbruck, Austria

Tel. +43 512 507 58 600

Email: [Andreas.Bernkop@uibk.ac.at](mailto:Andreas.Bernkop@uibk.ac.at)

# 1.1 Recovery yield tuftsin

The recovery yield of tuftsin in methanol was determined to validate the successful extraction of the peptide in this organic solvent. Therefore, HIP was dissolved in SEDDS-1, SEDDS-2, and SEDDS-3 preconcentrates at a concentration of 20 mg/mL, 26 mg/mL, and 30 mg/mL, respectively. Aliquots of 10 µL were diluted 1:9 with methanol and the concentration of tuftsin was quantified via HPLC. The recovery yield was calculated using Equation (S.1):

$recovery yield tuftsin \left( \% \right)= \frac{c_{tuftsin quantified via HPLC}}{c_{tuftsin initially dissolved in SEDDS preconcentrate}} \times100$ (S.1)


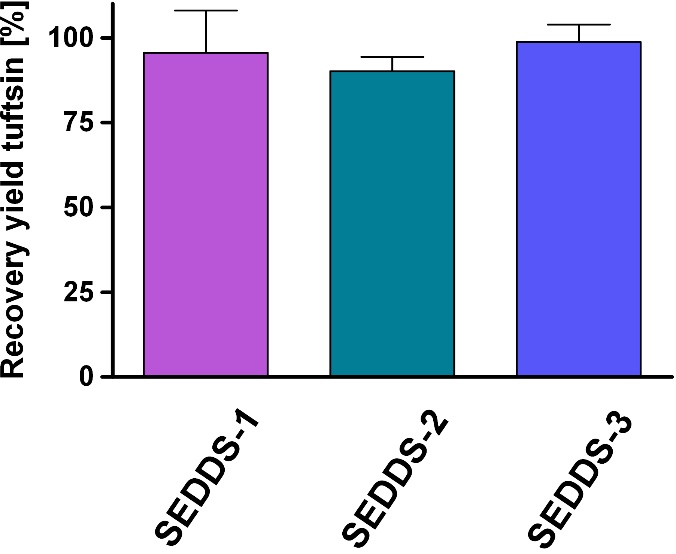


**Fig. S1** Recovery yield [%] of tuftsin extracted from SEDDS preconcentrates using methanol. Samples are indicated as follows: SEDDS-1 (    ), SEDDS-2 (    ) and SEDDS-3 (    ). Indicated values are means ± standard deviation (n ≥ 3).

The dilution of tuftsin-loaded SEDDS preconcentrates with methanol resulted in the recovery of 96% (w/w) of tuftsin from SEDDS-1, 90% (w/w) from SEDDS-2 and 99% (w/w) from SEDDS-3 in the methanol phase that was injected into the HPLC system. The polar structure of tuftsin, which bears two positive charges located on the amino groups, indicates a high degree of solubility in polar solvents such as methanol. Furthermore, several methods for detecting tuftsin use methanol as a solvent [1, 2] and previous studies investigating the incorporation of tuftsin into liposomes also employed methanol for dissolving tuftsin [3, 4]. These findings underscore the overall high solubility of tuftsin in this particular organic solvent. Consequently, it can be concluded that methanol is an efficient solvent for the detection of tuftsin in mixtures of excipients.

# 1.2 Impact SEDDS-3 on aminopeptidase N activity

To further investigate the inhibitory effect of SEDDS-3 on aminopeptidase N activity, citronellol and caprylic acid were tested separately. To solubilize these lipophilic components in 20 mM HBS pH 6.5, PEG-35 castor oil was added as it is a component of the SEDDS-3 formulation itself. Caprylic acid and PEG-35 castor oil were homogenized in 20 µL each on a Thermomixer at 60°C and 2000 rpm for 1h. Similarly, citronellol and PEG-35 castor oil were homogenized in 10 µL and 20 µL, respectively. Both mixtures were dissolved in HBS containing 5mM *L*-Leucine-4-nitroanilide to reach a final sample volume of 500 µL. To initiate the enzymatic reaction, 500 µL of a 41.6 mU/mL aminopeptidase N solution in HBS were added to each sample. The samples were incubated on a thermomixer for 1 h at 37 °C and 400 rpm agitation. To halt the enzymatic reaction, 1 mL of methanol containing 2% (v/v) TFA were added to each sample. Subsequent to centrifugation at 12,500 rpm, the concentration of 4-nitroaniline in the supernatant was quantified and the inhibitory activity was calculated using Equation (5). A 5 mM substrate solution served as the positive control, representing 100% enzyme activity. Inhibitory activities of SEDDS-3, PEG-35 castor, caprylic acid and citronellol are illustrated in Fig. S2.


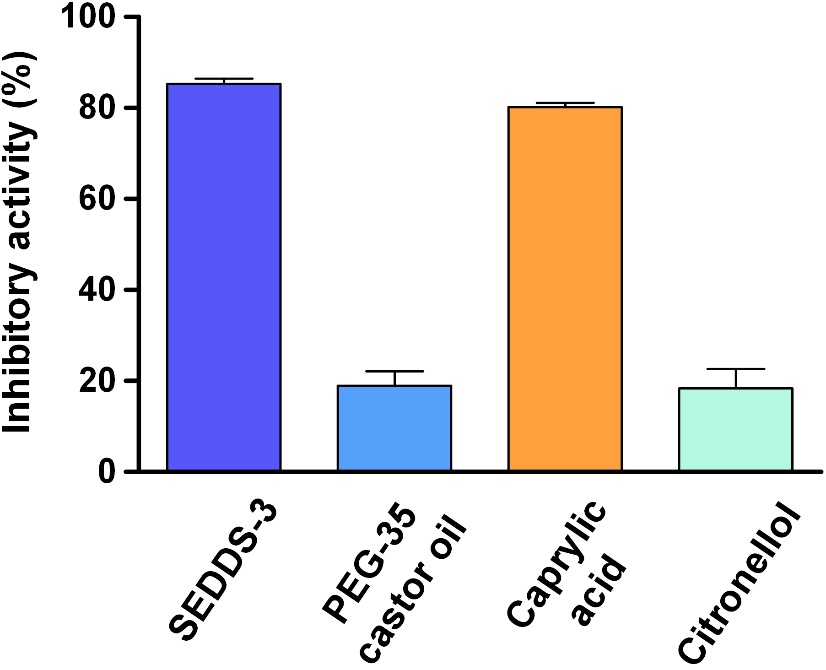


**Fig. S2** Inhibitory activity [%] of SEDDS-3 (    ), PEG-35 castor oil (    ), caprylic acid (    ) and citronellol (    ) towards aminopeptidase N. Data are presented as means ± standard deviation (n ≥ 3).

SEDDS-3 demonstrates an inhibitory effect on aminopeptidase N of 85%. In comparison, the individual excipients PEG-35 castor oil as surfactant and citronellol as co-solvent exhibit a significantly lower effect on the enzyme activity with 19% and 18% inhibition, respectively. Notably, the lipid component caprylic acid demonstrates a significantly stronger inhibitory activity of 80%. These findings provide substantial evidence that the inhibitory effect of SEDDS-3 is predominantly attributed to caprylic acid.

# 1.3 References

1. Murugesan K, Srinivasan P, Mahadeva R, Gupta CM, Haq W (2020) Tuftsin-Bearing Liposomes Co-Encapsulated with Doxorubicin and Curcumin Efficiently Inhibit EAC Tumor Growth in Mice. Int J Nanomedicine Volume 15:10547–10559. https://doi.org/10.2147/IJN.S276336

2. Siddiqui MZ, Sharma AK, Kumar S (1996) Solution conformation of tuftsin. Int J Biol Macromol 19:99–102. https://doi.org/10.1016/0141-8130(96)01108-7

3. Khan MA (2006) Toxicity, stability and pharmacokinetics of amphotericin B in immunomodulator tuftsin-bearing liposomes in a murine model. Journal of Antimicrobial Chemotherapy 58:125–132. https://doi.org/10.1093/jac/dkl177

4. Khan MA, Nasti TH, Saima K, Mallick AI, Firoz A, Wajahul H, Ahmad N, Mohammad O (2004) Co-administration of immunomodulator tuftsin and liposomised nystatin can combat less susceptible *Candida albicans* infection in temporarily neutropenic mice. FEMS Immunol Med Microbiol 41:249–258. https://doi.org/10.1016/j.femsim.2004.03.011
